# Portable Optical Epidural Needle-A CMOS-Based System Solution and Its Circuit Design

**DOI:** 10.1371/journal.pone.0106055

**Published:** 2014-08-27

**Authors:** Cihun-Siyong Alex Gong, Shih-Pin Lin, M. Susan Mandell, Mei-Yung Tsou, Yin Chang, Chien-Kun Ting

**Affiliations:** 1 Department of Anesthesiology, Taipei Veterans General Hospital and National Yang-Ming University, Taipei, Taiwan; 2 Department of Electrical Engineering, School of Electrical and Computer Engineering, College of Engineering, Chang Gung University, Taoyuan, Taiwan; 3 Portable Energy System Group, Green Technology Research Center, College of Engineering, Chang Gung University, Taoyuan, Taiwan; 4 Department of Anesthesiology, University of Colorado Health Sciences Center, Aurora, Colorado, United States of America; 5 Institute of Biomedical Engineering, National Yang-Ming University, Taipei, Taiwan; San Raffaele Scientific Institute, Italy

## Abstract

Epidural anesthesia is a common anesthesia method yet up to 10% of procedures fail to provide adequate analgesia. This is usually due to misinterpreting the tactile information derived from the advancing needle through the complex tissue planes. Incorrect placement also can cause dural puncture and neural injury. We developed an optic system capable of reliably identifying tissue planes surrounding the epidural space. However the new technology was too large and cumbersome for practical clinical use. We present a miniaturized version of our optic system using chip technology (first generation CMOS-based system) for logic functions. The new system was connected to an alarm that was triggered once the optic properties of the epidural were identified. The aims of this study were to test our miniaturized system in a porcine model and describe the technology to build this new clinical tool. Our system was tested in a porcine model and identified the epidural space in the lumbar, low and high thoracic regions of the spine. The new technology identified the epidural space in all but 1 of 46 attempts. Experimental results from our fabricated integrated circuit and animal study show the new tool has future clinical potential.

## Introduction

Epidural analgesia is a common but technically challenging method for providing pain relief [1], [2]. Care providers use anatomic landmarks and tactile information to locate a space that is only 2–7 mm in diameter [2]. The space is detected as a loss of resistance to pressure exerted on a syringe barrel coupled to a needle that travels through the patient’s tissue planes [2]. This requires the provider to sense differences between ligament tissue tone and the epidural space while blindly advancing a fluid or air filled syringe. Anatomic variation between patients and the experience of the provider play a large role in how well an epidural works [3], [4]. Inability to correctly locate the epidural space is one of the common reasons for the 5–10% failure rate reported in the literature [5]. It can also lead to complications including dural puncture with resulting post-dural puncture headache or spinal nerve injury.

We previously reported successful identification of the epidural space in real time using a novel system developed in our laboratory [6], [7]. We tested if optical probes mounted inside a standard Tuohy needle could identify the epidural space in a porcine model. Our optical system performed well. Dual wavelength optical impulses (650 and 532 nm) reliably identified the ligamentum flavum. Because the ligamentum flavum is the entry point to the epidural space (farthest tissue plane from the cerebrospinal fluid), the approach improves the safety of the instrument. The fidelity of the optical signals was sufficient to implement an intelligent recognition system for needle placement [8]. The primary limitation of our optical model was the large cumbersome setup with a long electrical connection coming out of the needle and connection to a computer.

Our laboratory therefore used novel technology to develop a more compact and mobile optical guided probe for practical clinical use. We used a complementary metal oxide semiconductor (CMOS) to construct a miniature optical probe. The CMOS is a type of digital circuitry in chip technology. It allows for a high density of logic functions and is therefore more compact than other forms of integrated technology. The immunity to noise and low static power consumption makes this an ideal technology to use for optic guided epidural needle insertion.

We questioned if the smaller probe performed as well as our previous fiberoptic-guided needle. The probe was therefore tested in the same *invivo* porcine model used in our original study. We aimed to determine if the optic signals from CMOS technology could reliably identify the tissue planes during needle passage into the epidural space. The electronic circuitry of our novel probe construction is described.

## Methods

### Porcine model

Our study was approved by the Institutional Animal Care and Use Committee of Taipei Veterans General Hospital. We conducted invivo studies in four Duroc and Landrance pigs each weighing approximately 25 kg. General anesthesia was induced with intramuscular injection of Tiletamine-zolazepam (5 mg/kg) and maintained with isoflurane (1.5–2%) through an endotracheal tube during the whole course of study. Animals were then placed in the left lateral position for epidural needle insertion. Vital signs were continuously monitored during the procedure.

A standard 17 G Tuohy needle with newly designed miniature optical stylet were inserted into the lumbar and then low and high thoracic segments of the porcine vertebral column. The needle was advanced until the alarm sounded because the signal indicated entry into the epidural space. Approximately 10–12 punctures at different spinal levels were performed in each pig. Electronic signals from the 650 nm wavelength were retrieved for analysis. To test the accuracy of our system, the stylet was removed when optic properties indicated the needle tip was in the epidural space. A catheter was then inserted and placement confirmed using ultrasonography (Vivid e, GE healthcare, London, United Kingdom). Radiography following injection of the catheter with contrast medium was then done as a second confirmation that the needle entered the epidural space.

### Statistical Analysis

Parametrical data are presented as the mean±SD. Linear mixed model analysis did not identify any variation in the optical signal at different levels along the vertebral column in our previous study [7]. The optical characteristics of the tissue were consistent regardless of axial position on the spine, a naïve pooled technique was used to put all data together from different spine level for analysis statistically. A paired *t* test was used to compare the signal amplitude of 650 nm between the epidural space and the ligamentum flavum. The results were considered to be statistically significant for *P* values less than 0.05. A receiver operating characteristic curve (ROC) was used to determine the optimal cutoff values for signal amplitudes of 650 nm to differentiate the epidural space from the ligamentum flavum. Area under the ROC curve with its 95% CIs was also calculated to evaluate the predictive validity of 650 nm All statistical analyses were performed with SPSS software (v.17; SPSS Inc., Chicago, IL).

### Optical Needle

The optic system has been previously described [6], [7]. In brief, a fiberoptic bundle was inserted into a hollow stylet of a standard 17 G Tuohy needle (Arrow, Teleflex Incorporated, Limerick PA). The fibers were sealed into a single bundle, manually beveled to fit the shape of the Tuohy needle tip and inserted into a hollow stylet. Our previous studies used two wavelengths (532 and 650 nm) to test the optic system. The 532-nm wavelength did not perform as well as the 650 nm wavelength in our previous invivo study [6], [7]. We therefore only used 650 nm wavelength in this study to determine how new CMOS-chip technology influenced signal recovery. An Avalanche photodiode (S5345 Hamamatsu Photonics, KK Shizuoka, Pref Japan) converted the optic to an electrical signal. The signal was amplified so it could be read by the operator. In our new system, an optical impulse triggered alarm was added to inform the user when the epidural space was detected. The threshold of the alarm was set using the reflection signals of ligamentum flavum and epidural space from our previous study results.

### The CMOS chip

**Figure 1:**
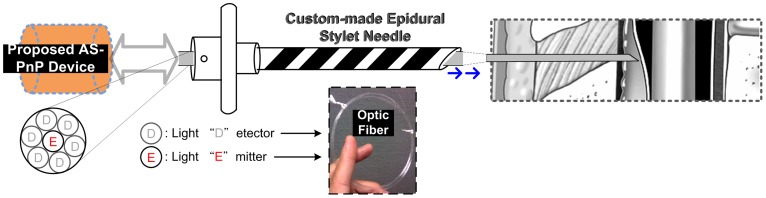
Conceptual drawing of the optic system.

**Figure 2:**
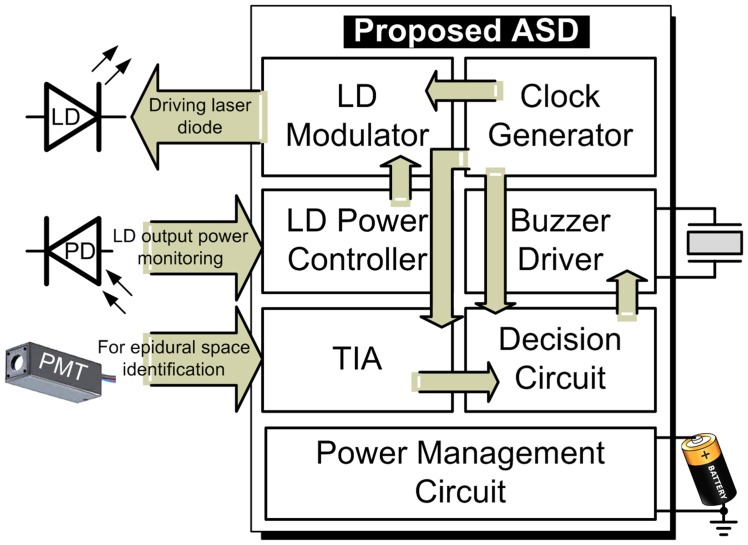
Proposed application-specific device for the optical needle placement.

We modified our previously described fiberoptic epidural stylet in order to produce a compact instrument more suitable for mobile clinical use. The instrument was composed of a fiberoptic light source that is translated into a visual signal and linked to an alarm indicating location of the target tissue. The components of our epidural needle were integrated into a single chip using solid-state electronics. The conceptual drawing of this work is shown in Fig. 1. We therefore used an application-specific device (Fig. 2). In this system, a chip with different sub-circuits performed the same signal processing as our previous off-the-shelf discrete components.

#### Overview of the System

Advanced Semi Conductor Development (ASD) was used to miniaturize the optic system. This is a highly integrated semiconductor using distinct building blocks. In our system, the off-ASD laser diode (LD) used for the Light Emitter was driven by the LD Modulator block (Fig. 2). The Clock Generator Block modulated signals required by the enabled LD Modulator. The LD Power Controller dominated the average optical output power of the LD through a feedback loop formed by the LD. The loop consisted of the photodiode (PD) used for sensing the light emitted from the LD, the LD Modulator block, and the LD Power Controller.

The Light Detectors shown in Fig. 1 received the tissue-reflected signals for post processing. There were two stages in signal readout: the signal was first amplified through a photomultiplier tube (PMT) and then processed through a transimpedance amplifier (TIA). Of the three options for PMT readout; the “gated integrator”, the “continuous current”, and the “photo counting”, we chose the gated integrator for our laser application because the arrival times of the periodic PMT output current pulses were known.

**Figure 3:**
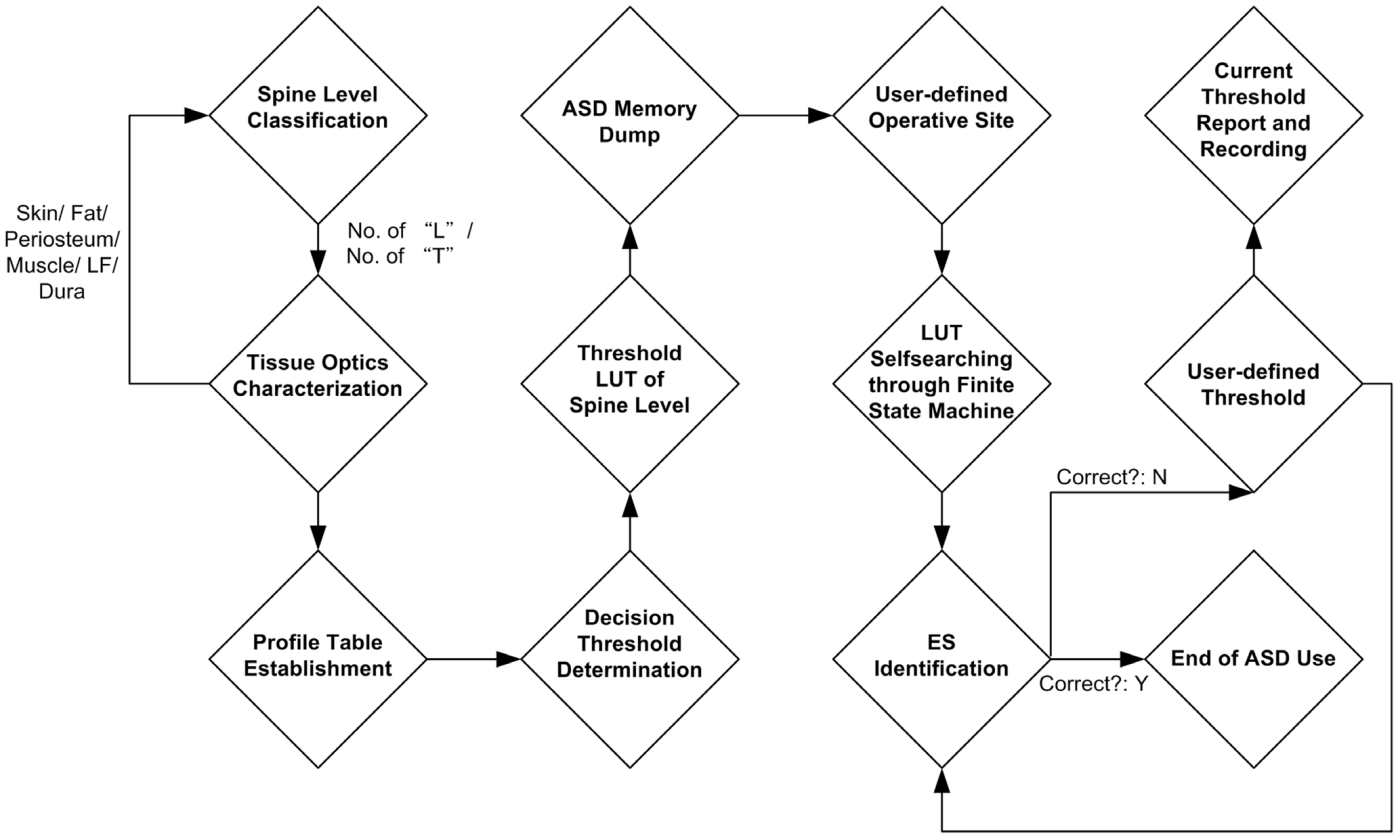
Standard operation procedure of proposed assistive system.

The output of the TIA was linked to a clock signal through a blend circuit to generate a driving signal (buzzer) for an alarm. The audio alarm was added to alert the clinician that the target point (epidural space) was reached. The target point was identified when signals from the ligamentum-flavum-reflected light was lost. The system was battery-powered and its supply regulated by the Power Management Circuit block. The standard operation procedure of the implemented epidural space (ES) identification prototype is illustrated with Fig. 3.

## Results

There were a total of 46 punctures recorded in 4 pigs. The epidural space was identified using the optical impulses. Ultrasound and contrast studies showed the epidural catheter was correctly placed in 45 attempts, indicating a 97.82% success rate (Table 1).

**Table 1:** Results of Animal Study.

Pig #	1	2	3	4
Spine level				
**L4-5**	O	O		O
**L3-4**	O		O	O
**L2-3**	O	O		O
**L1-2**	O	O	O	O
**T14-L1**		O	O	
**T13-14**	O	O	O	O
**T12-13**	O	O	O	O
**T11-12**	O		X	O
**T10-11**	O	O	O	O
**T9-10**		O	O	O
**T8-9**	O	O	O	O
**T7-8**	O	O		O
**T6-7**	O	O	O	O
**T5-6**		O	O	O

O: Successful, X: Fail.

**Figure 4:**
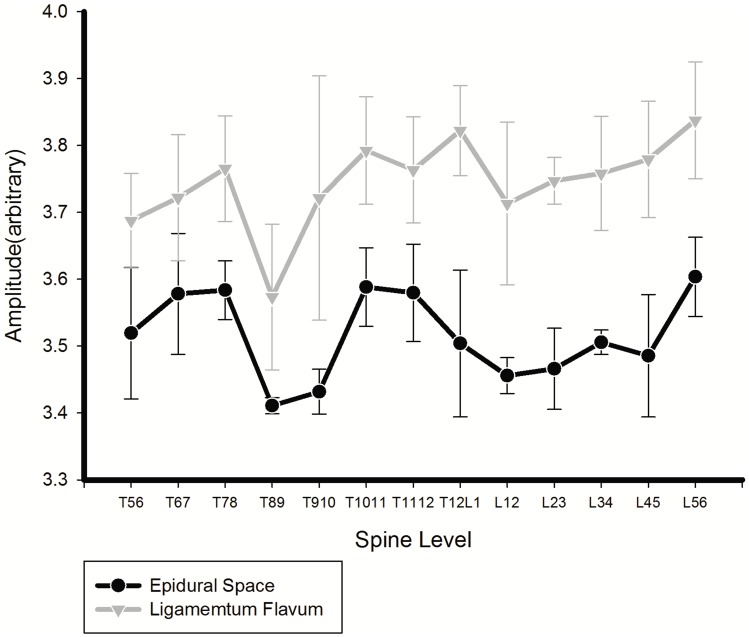
The reflective amplitude at different spinal levels in the porcine model (mean+/SD).

**Figure 5:**
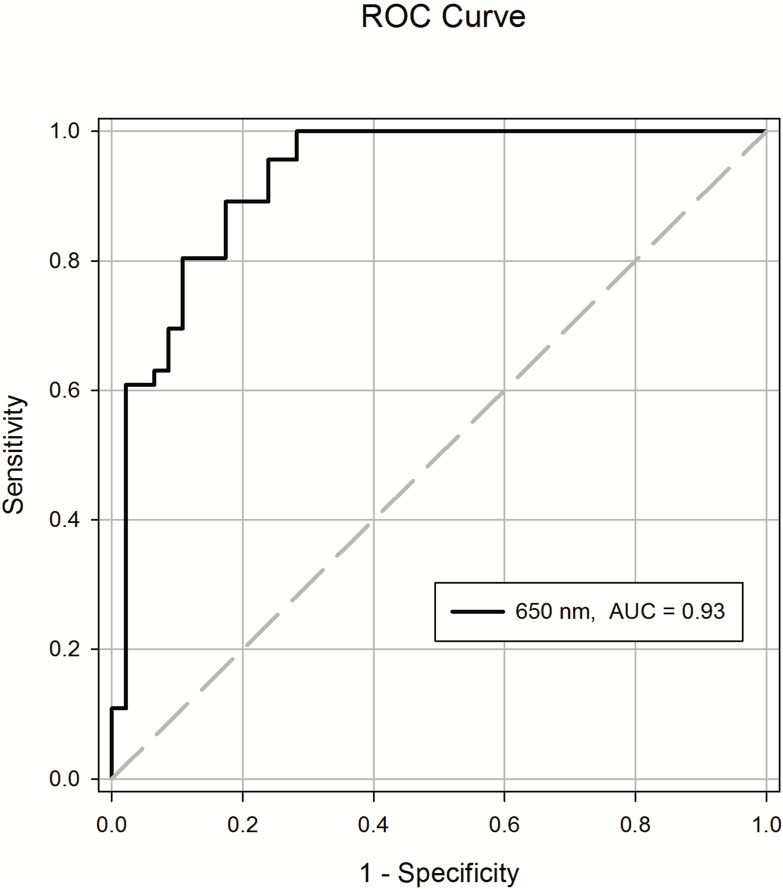
The receiver operating characteristic (ROC) cure of observations from epidural puncture in the porcine model. The area under curve for 650 nm is 0.93.

Fig. 4 illustrates a total of 46 needle insertions that were performed in the four pigs. The averaged magnitude of the reflective signals at the epidural space was 3.516+/0.093 at 650 nm, and at the ligamentum, it was 3.743+/0.117. A paired *t* test showed significant differences between the averaged magnitudes for the light reflected from the ligamentum flavum and the epidural space in the 650 nm wavelengths (*P<*0.001). ROC analysis verified the difference in optical properties between the ligamentum flavum and epidural space at wavelengths of 650 nm based on the sensitivity and specificity [9], [10]. Fig. 5 shows the ROC curve of the test. The test showed good discrimination between the epidural space and ligamentum flavum (Table 2). The area under curve for 650 nm was 0.927 (95% CI 0.874–0.980).

**Table 2:** Receiver Operating Characteristic Curve Analysis.

	Mean	SD	SEM	95%CI	P-value
LF	3.743	0.117	0.017		
ES	3.516	0.093	0.014		
AUC	0.927		0.027	0.874–0.980	<0.001

LF: Ligamentum flavum; ES: Epidural Space; AUC: Area under curve; CI confidence interval.

## Discussion

Our studies showed that optical analysis of tissue planes can be successfully miniaturized for use in clinical medicine using chip technology. The miniaturized system replicates the findings obtained from our previous optic analysis. This suggests that new chip technology can facilitate a number of logical functions needed for optical resolution of tissue planes that previously required large and bulky equipment. The miniature version of our optical device is compact and portable and is therefore more suitable for clinical use.

In our previous study, we showed that reflective light could be used to guide epidural placement [6], [7]. There were more successful epidural placements using the optical system compared to experienced operators [11], [12]. The reliable reflective optical impulses from the ligamentum flavum and epidural space provide more consistent information for guiding needle placement compared to tactile techniques. Only one person is required for optic guided needle placement. However, the equipment needed for optical placement was large and not easily portable. These are important design limitations. Therefore, we designed a small and portable version of our optical device by the selective use of chip technology.

There are two common problems leading to failed epidural placement. First is identification of an accurate trajectory leading from the skin to the ligamentum flavum. Second is placement of the needle tip into epidural space without passing through the dura mata. Our device was built to identify the epidural space and prevent accidental dural punctures. It does not identify needle trajectory for guiding purpose. However, surface 2D ultrasound can be used to estimate an accurate trajectory from the skin to epidural space [13].

We tested the reliability of our new optical system using our older system as a standard for performance. Our previous model identified the epidural space in all passages of the optical needle [6], [7]. The average magnitude of the reflective signals at the epidural space was similar between our previous system (3.565±0.194) and our new device (3.516+/0.093). A similar concordance was noted for the reflective signals at the ligamentum flavum (3.842±0.191) in our previous and new system (3.743+/0.117). Similar to our previous system, there were no significant differences in the optical characteristics of tissues at different levels of the spinal column [7]. Thus, there is no need to adjust the optical settings at different levels of epidural insertion.

The difference between the average magnitudes for the reflected light of the epidural space and ligamentum flavum was significant for our new miniaturized and original devices. This indicates that the optic signals can discriminate between the two anatomic structures without overlap. The ROC analysis verified the differences in optical properties between the two anatomic structures. The distinct differences in reflective optical patterns of the epidural space and ligamentum flavum may account for the higher degree of accuracy when placing the epidural needle compared to the average 10% failure rate using conventional tactile techniques [5], [14].

One of the possible errors in epidural placement can occur during interpretation of the optical signals. We have previously shown that reflective light impulses can be analyzed using discriminate analysis to identify the epidural space [8]. The data were tested using linear discriminate analysis to “decide” if the needle had entered the epidural space. However the discriminate analysis only had 80% sensitivity and specificity.

We therefore used a clock signal linked to a buzzer in our new system to alert the operator that the needle was within the epidural space. We linked the output of the transimpedance amplifier to a clock signal through a blend circuit to drive a signal to alert the operator that the signals from the ligamentum flavum-reflected light was lost. Using this technique there was only one failed attempt. We reason the failure is due to limitations in the standard operation procedure of the assistive system. The system alarmed when the needle tip passed into a pseudo-space before the ligamentum flavum. The pseudo-space has the same fiberoptic qualities as the epidural space. However, ligamentum flavum signals were not observed prior to entry. This indicates that a new assistive design is required to incorporate more complex information that sequentially identifies the ligamentum flavum and epidural space (loss of signal).

The decision threshold plays a key role in successful identification. The determination of threshold involves a choice of statistical methods. Currently, a small sample size is the primary problem to make this determination. We are currently working on ways to improve the performance of our standard operation procedure. The system requires a decision maker that simultaneously learns from large amount of unlabeled data, together with labeled data, and sets a reliable threshold. We are examining different ways to achieve this. The accuracy of the optical system should also be modified. Semiconductor technology suffers process variations which result in device imperfection. This affects the accuracy of light reception and degrades signal conditioning performance. The next-generation design will focus on adding a better on-system automatic calibrator to adjust itself after startup.

Our device is simple, sensitive and portable. Further, the data provides recordable information for review and quality improvement. Direct observation of the reflected light using an oscilloscope may also facilitate training because both the student and instructor can observe the same information simultaneously. One of the limitations of our technique observed in our previous study was that the amplitude of the reflective light was influenced by the direction of the needle bevel. However, this influence became minimal with greater use of the optic guided needle indicating that there was a learning curve required for efficient use [6]–[8].

The findings of our study are derived from an animal model and we did not test our devise in a human population. We anticipate the optical characteristics of human epidural space are similar. The tissue composition and anatomic order of tissue planes are identical. We were able to compensate for anatomic differences based on size of the study animal. The distance from the ligamentum flavum to the dura was 2 to 4 mm in the lumbar and thoracic of piglet. It varied with size and weight of piglet. These variations did not affect the outcome of our study. In conclusion, our optical epidural needle is a reliable method. The device can decrease failed epidural catheters. By taking the advantage of CMOS widely used in many advanced and miniaturized medical applications [15], our new ASD is compact and portable.

### Addendum (System Specifications and Rationale)

In this section, we describe the system in detail, outlining our rationale for circuit design.

#### Control of optical power (Fig. S1)

We controlled average optical power to prevent instrument damage by comparing gate voltage of the transistor MP7 (the produced current ipd from the incident light of the PD), against the reference voltage Vref1. The resistor R1 and capacitor C1 are frequency compensation components used to assure stability of the LD Power Controller.

#### LD modulator schematics (Fig. S2)

Minimal threshold current, *ith* was needed to properly drive the LD [16]. We chose a transistor with off-chip bias tuning instead of an on-chip constant bias for better temperature coefficient compensation. The feedback power control current was generated by the MN5-R2 path through a unity-gain buffer formed by the two transistors MN3 and MN4. The source-coupled circuit constructed by the MN6, MN7, and MN8 supplied the total current producing maximal optical power modulation. Vref2 drove the MN7 to provide half the current. To prevent “ripple (residual variation on the Vmod provider/Clock Generator) we designed an active power regulator shown in Fig. S3. This prevented large transient current that exceeds the limit of the emitted optical LD optical power.

#### Power Management Circuit

The supply regulator (Power Management Circuit in Fig. 2) had three sub-building blocks (Fig. S3): 1. the battery-powered Voltage Reference with supply-independent reference current through the generated bias V ref and whose zero current mirroring condition could be broken by its Startup circuit; 2. the amplifier formed a negative feedback loop so low-ripple supply generation could be imposed by comparison with the Vref to control the power transistor Mp; 3. the feedback voltage divider network consisting of the passive components (Rc, Cc. etc.) used for on-the-fly generation of the Vfb associated with the ripple on the Vdd.

#### Clock Generator (Fig. S4)

We used output CLK-out to generate clock signals in demand for our ASD. The Vmod was obtained by processing the CLK-out in a low-pass filter followed by an AC-coupled circuit with its output biased at the Vdd. The low-pass filtering circuit assured an ASD safety window so the rising and the falling edges of the Vmod would be within the range protecting the LD from over light power. We noted the low-level supply Vdd2 offered a low-frequency generation with low-power consumption and was not an “unregulated” energy source. The power supply stabilization allowed the LD Modulator to drive the LD without damage.

The ring oscillator of the Clock Generator is shown in Fig. S4. The loop formed by the current-starved inverters and NAND circuit were sized to satisfy the Barkhausen criterion to start oscillation with an expected frequency at power-up. The sw from the combination of power-on-reset signal and special-purpose control signal activated/deactivated the Clock Generator. Transistors MP8–MP10 and MN9–MN11 were used as a low-power Schmitt trigger [17]. The signal comparison with hysteresis could reject its on-input noise to achieve pulse shaping. The circuit of the MP11–MP12 and MN12–MN13 functioned as level converters to other building blocks.

#### TIA and the Decision Circuit building blocks (Fig. S5)

The output signal of the PMT was fed into a clocked integrator where the input is synchronous sampled by the CMOS switch (gated by CLK-out). The amplified signal representing the reflected light from the tissue was integrated and unity-gain buffered in time to be processed. Once the acceptable level of the charge accumulated on the C1, it was sampled and subtracted from the previously sample through the controlling of the t1 and t2. The resulting value was compared with the Threshold, which can be set by the user to better identify the target. All the clock signals shown in the timing diagram of Fig. S5 were generated by the on-chip Timer. The operational amplifiers (Fig. S5) were placed with the same structures as shown in Fig. S3(c) with the exception of transistor sizing and offset nulling technique.

Offset Nulling was used to calibrate each of the amplifiers involved to minimize the process-and-fabrication-induced mismatch between the differential pairs. This is standard practice in modern semiconductor design [18]. This improves decision making. Circuit detail of the offset nulling technique is shown in Fig. S6(a). The transistors separately controlled by the Offset Nulling and biased at the Bias\_aux function as an auxiliary pair whose source terminals the Aux***_left_*** and Aux***_right_*** were connected with the counterparts of the amplifier input pair in parallel, respectively. Fig. S6(b) shows the circuit driving the buzzer (the Buzzer Driver) for target identification. Once the epidural space was identified the Decision Output was kept constant until the system was manually reset. The AND gate involved was able to control its output slew rate (current-starved design similar to the counterparts shown in Fig. S4) so that it did not cause ill effect in the buzz sound.

The ASD was realized in a 0.35-µm CMOS process. The fabricated chip for epidural space identification was tested using PCBs (Printed Circuit Boards), as shown in Fig. S7. The required test currents used to emulate the currents associated with the emitted light and the reflected counterpart were generated by a potentiostat/galvanostat, supplied with the EC-Lab, which supports programmable µA-level current generation. For practical use, it was powered by an energy source with three serially connected small-size 2.5-V end-point-voltage batteries to provide two unregulated power supplies: Vbat = 7.5 V and Vdd1 = 5 V. The regulated counterparts Vdd = 5 V and Vdd2 = 2.5 V were generated by the on-chip active regulator. The proposed ASD system consumes an average current of 65 mA in total, with approximately 60% in the LD-associated devices and circuits. The Buzzer Driver dominated the rest of the power consumption. An 1-KHz clock was generated from its building block with sub-microwatt average power dissipation. The system emitted an average light power of 3 mW to the targets and received reflected feedback of 4.5 µA in the PMT output at the ligamentum flavum and 0.7 µA at the epidural space. The effective bandwidth (−3 dB) of the TIA was set as 9 KHz to keep errors below 3% for an internal CLK out rising/falling time of approximately 40 us. An oscilloscope trace showing buffered output of the input of the Buzzer Driver can be seen from Fig. S8, which clearly demonstrates that the proposed design could ring an alarm when the epidural space was identified. An important factor indicating the ASD performance is the loop transient response versus power supply variation. The regulator was designed to prevent the ripple onto its output when a predetermined maximum output load of 80 mA was connected. The experimental regulator output performance is shown in Fig. S9 where an average of 250-mV peak-to-peak Vdd voltage variation was measured for the ASD.

## Supporting Information

Figure S1 **Circuit Schematic of the LD Power Controller.**
(TIF)

Figure S2 **Circuit Schematic of the LD modulator.**
(TIF)

Figure S3 **Schematic of the Power Management Circuit.**
(TIF)

Figure S4 **Design of the Clock Generator.**
(TIF)

Figure S5 **The proposed TIA and analog part of the Decision Circuit.**
(TIF)

Figure S6 (a) The offset nulling technique. (b) The Buzzer Driver circuit.
(TIF)

Figure S7 **Measurement setup of the fabricated ASD chip.**
(TIF)

Figure S8 **Measured buffered output of the Buzzer Driver input in an epidural space identification test.**
(TIF)

Figure S9 **Measured regulator output performance of our ASD.**
(TIF)
