# Supplementary figures and images for: Portable Optical Epidural Needle-A CMOS-Based System Solution and Its Circuit Design
Source: PLoS One. 2014 Aug 27;9(8):e106055. doi: 10.1371/journal.pone.0106055 (PMC4146568; doi:10.1371/journal.pone.0106055)

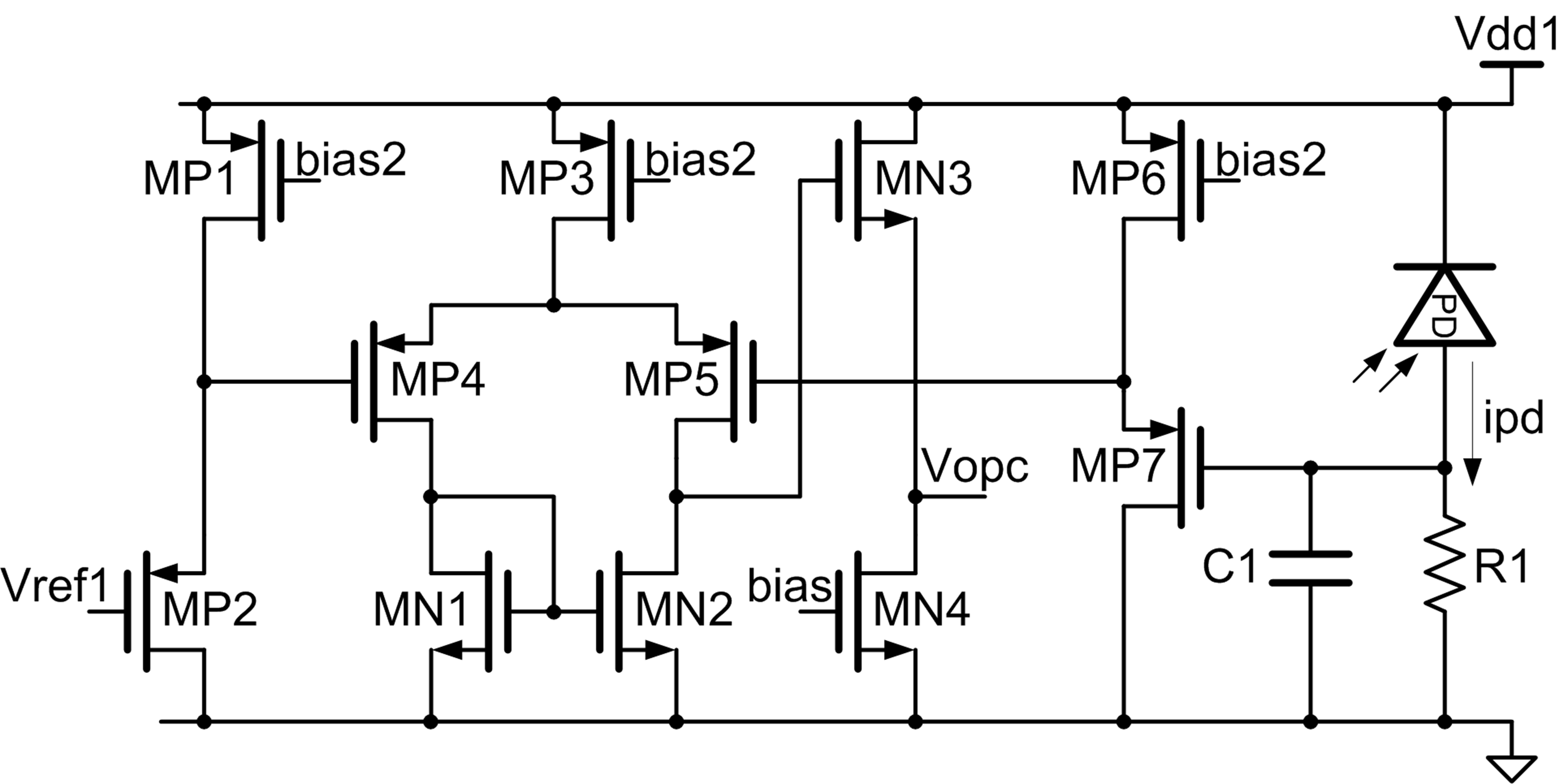

Supplement: Figure S1 — Circuit Schematic of the LD Power Controller. (TIF) [file pone.0106055.s001.tif]

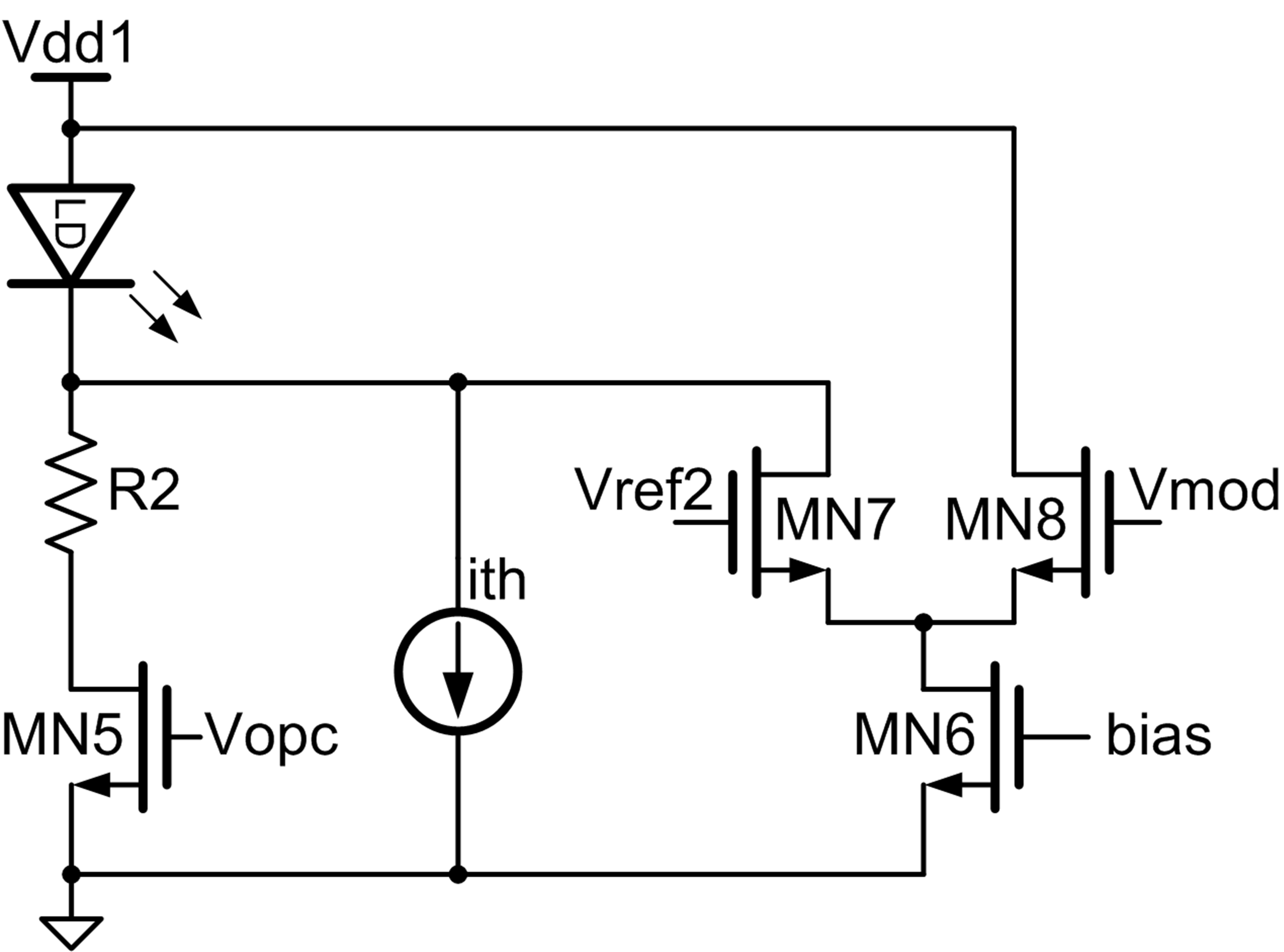

Supplement: Figure S2 — Circuit Schematic of the LD modulator. (TIF) [file pone.0106055.s002.tif]

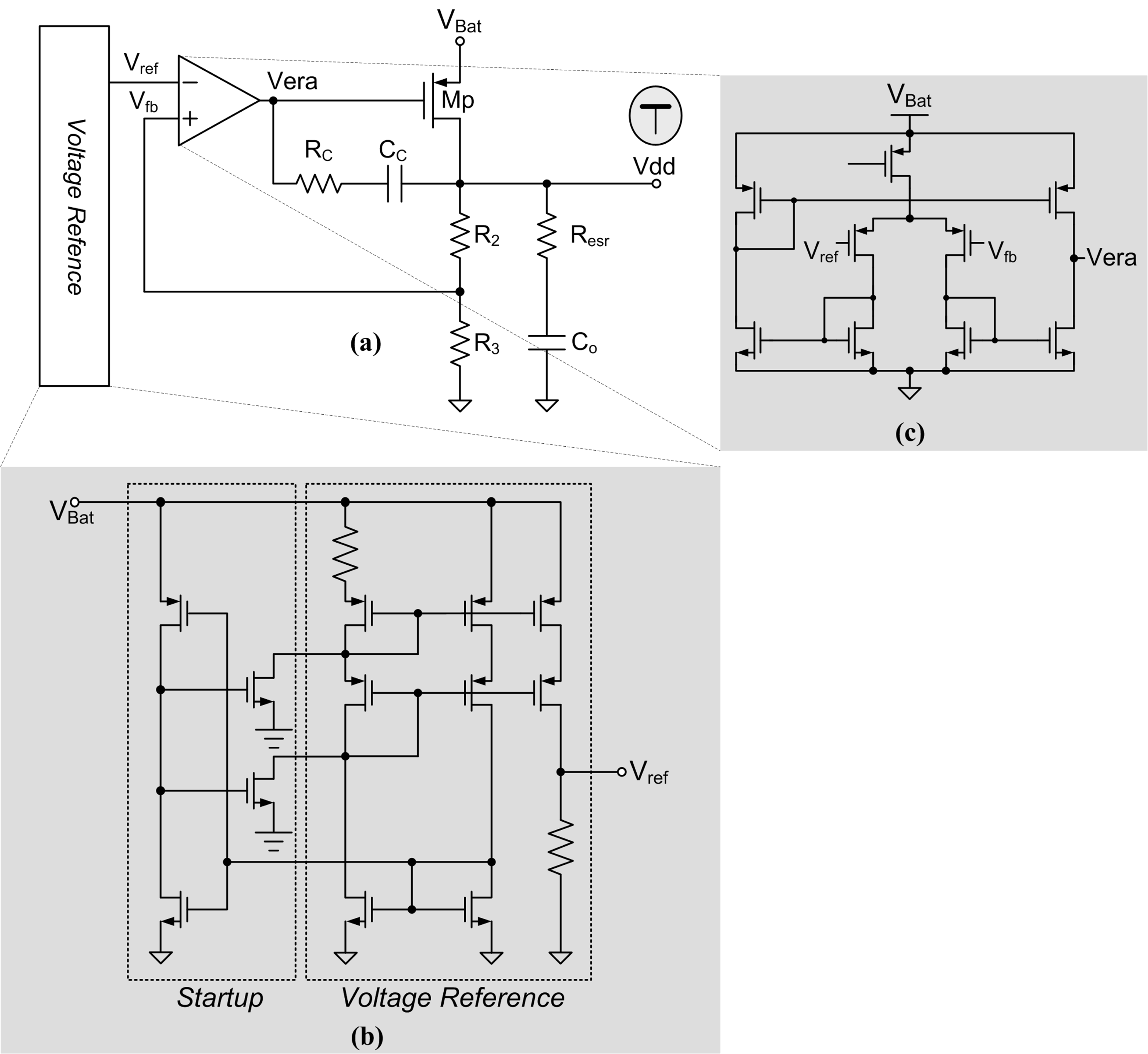

Supplement: Figure S3 — Schematic of the Power Management Circuit. (TIF) [file pone.0106055.s003.tif]

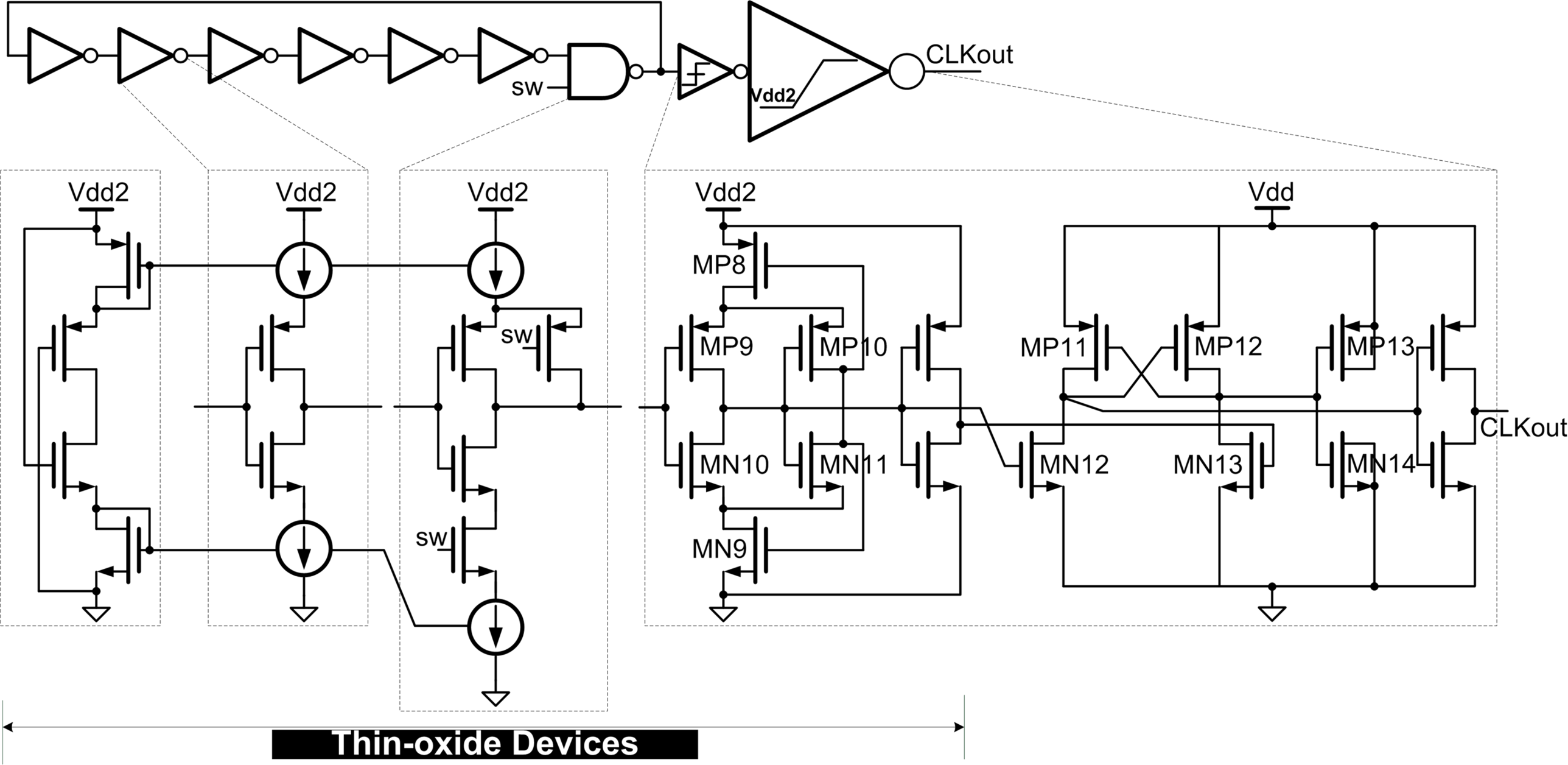

Supplement: Figure S4 — Design of the Clock Generator. (TIF) [file pone.0106055.s004.tif]

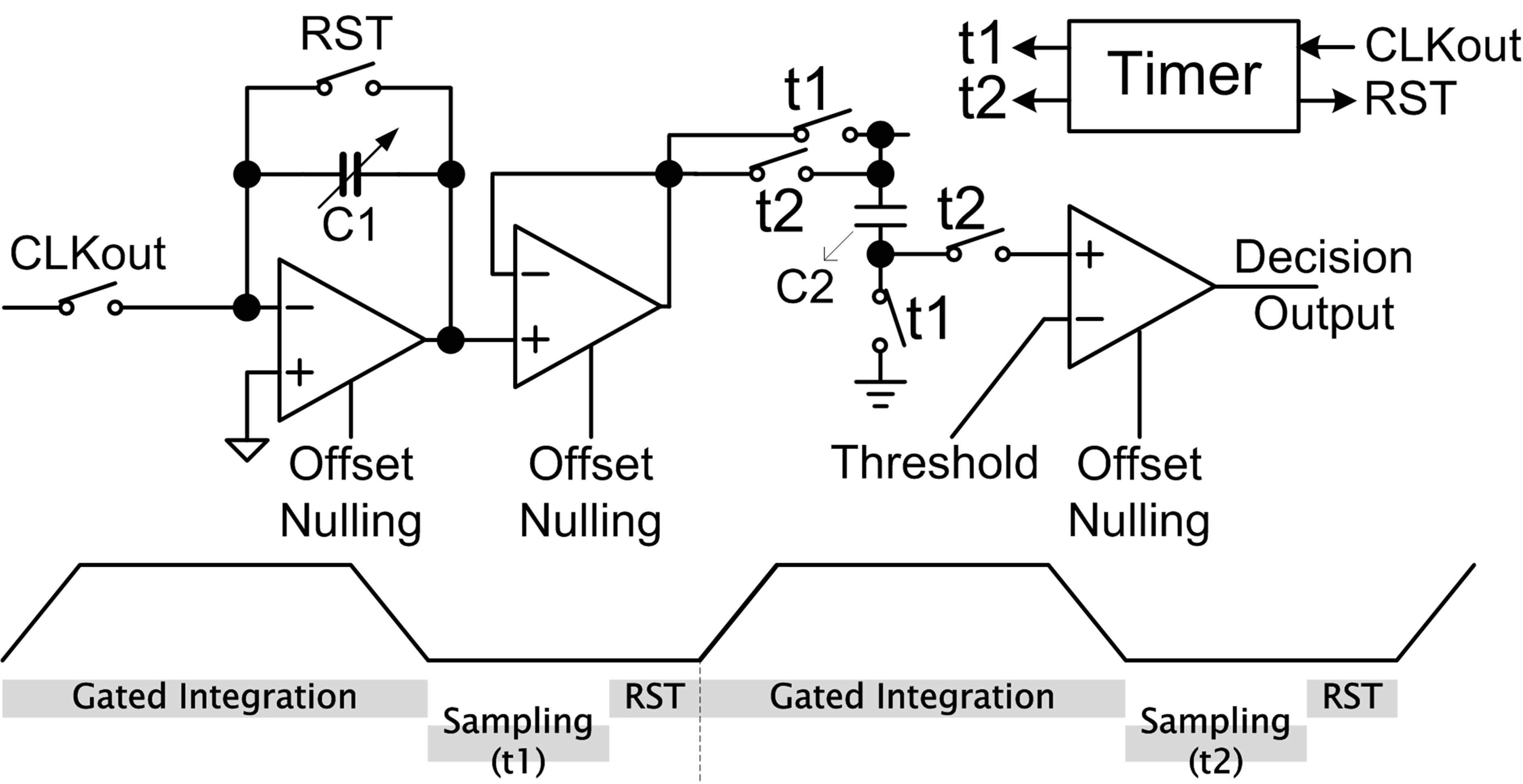

Supplement: Figure S5 — The proposed TIA and analog part of the Decision Circuit. (TIF) [file pone.0106055.s005.tif]

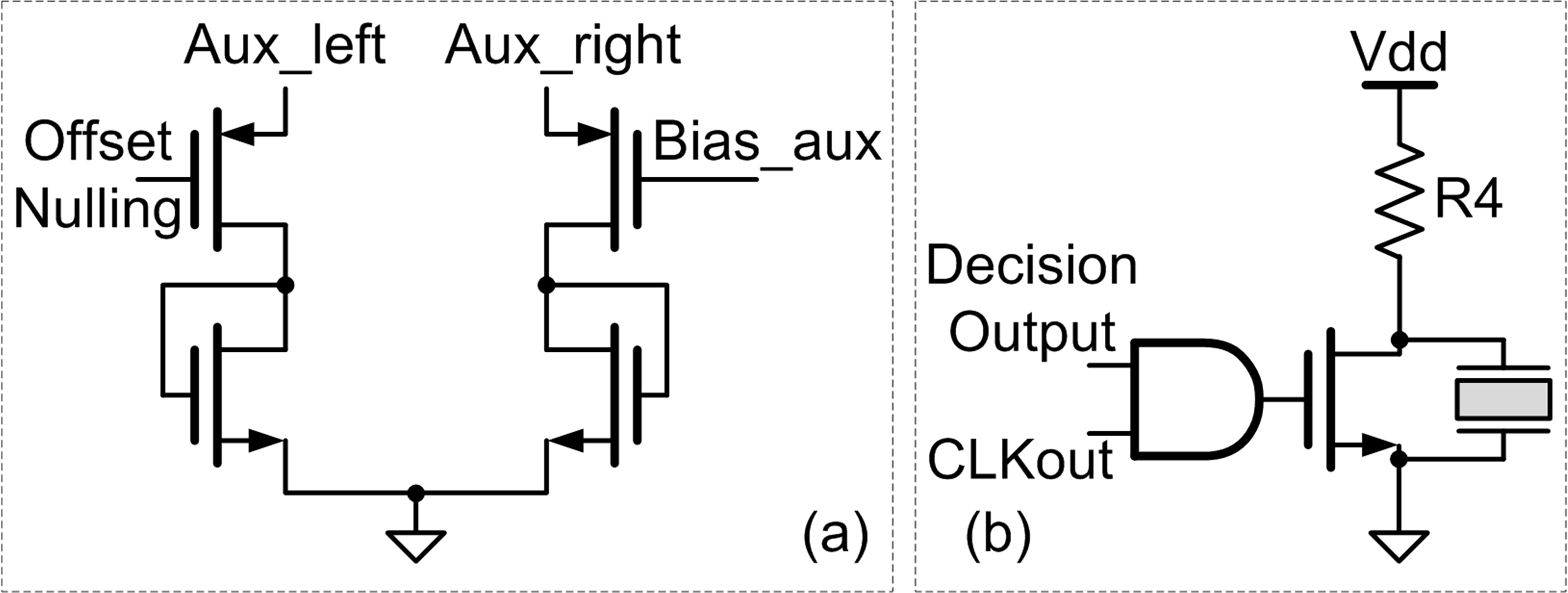

Supplement: Figure S6 — (a) The offset nulling technique. (b) The Buzzer Driver circuit. (TIF) [file pone.0106055.s006.tif]

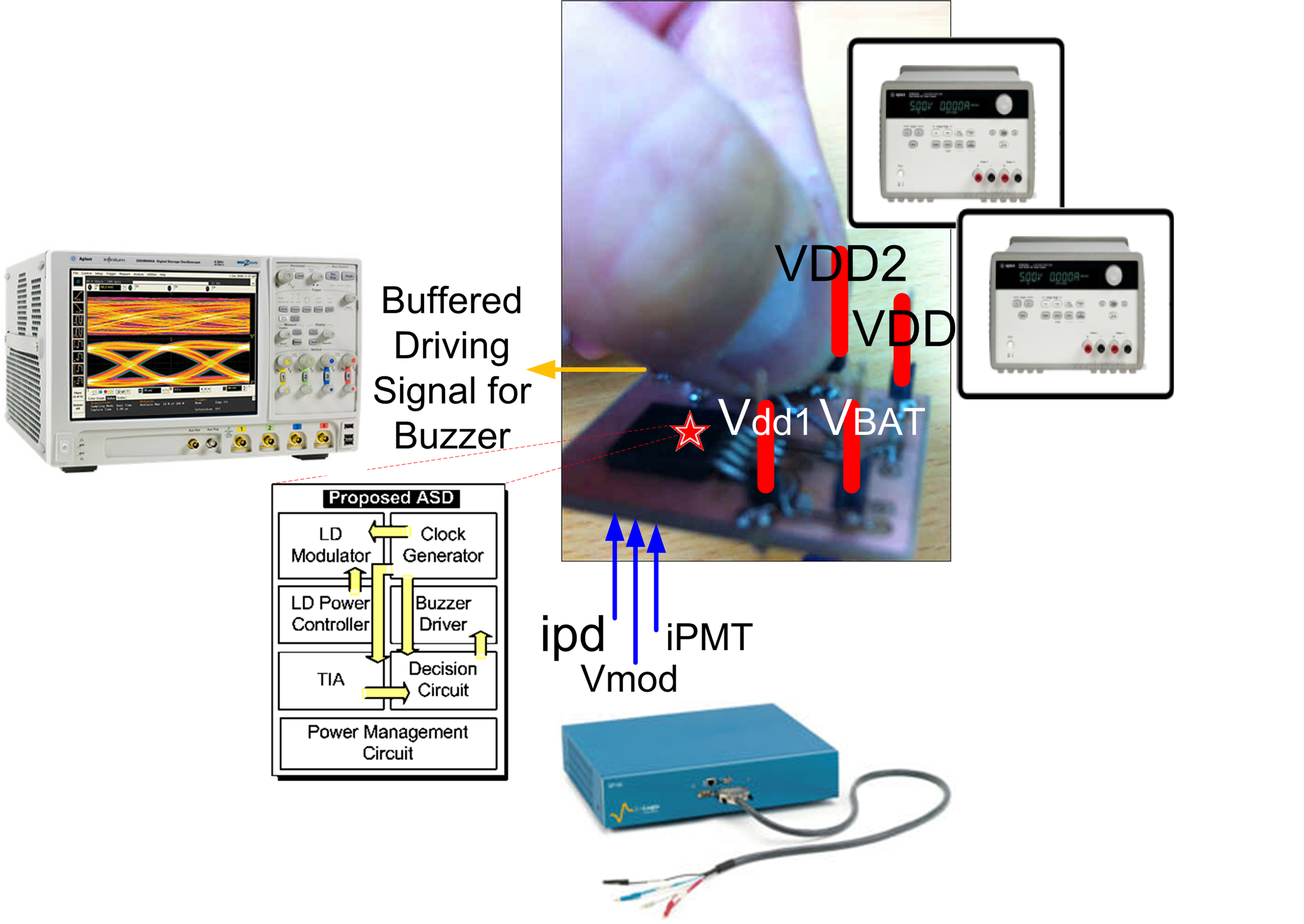

Supplement: Figure S7 — Measurement setup of the fabricated ASD chip. (TIF) [file pone.0106055.s007.tif]

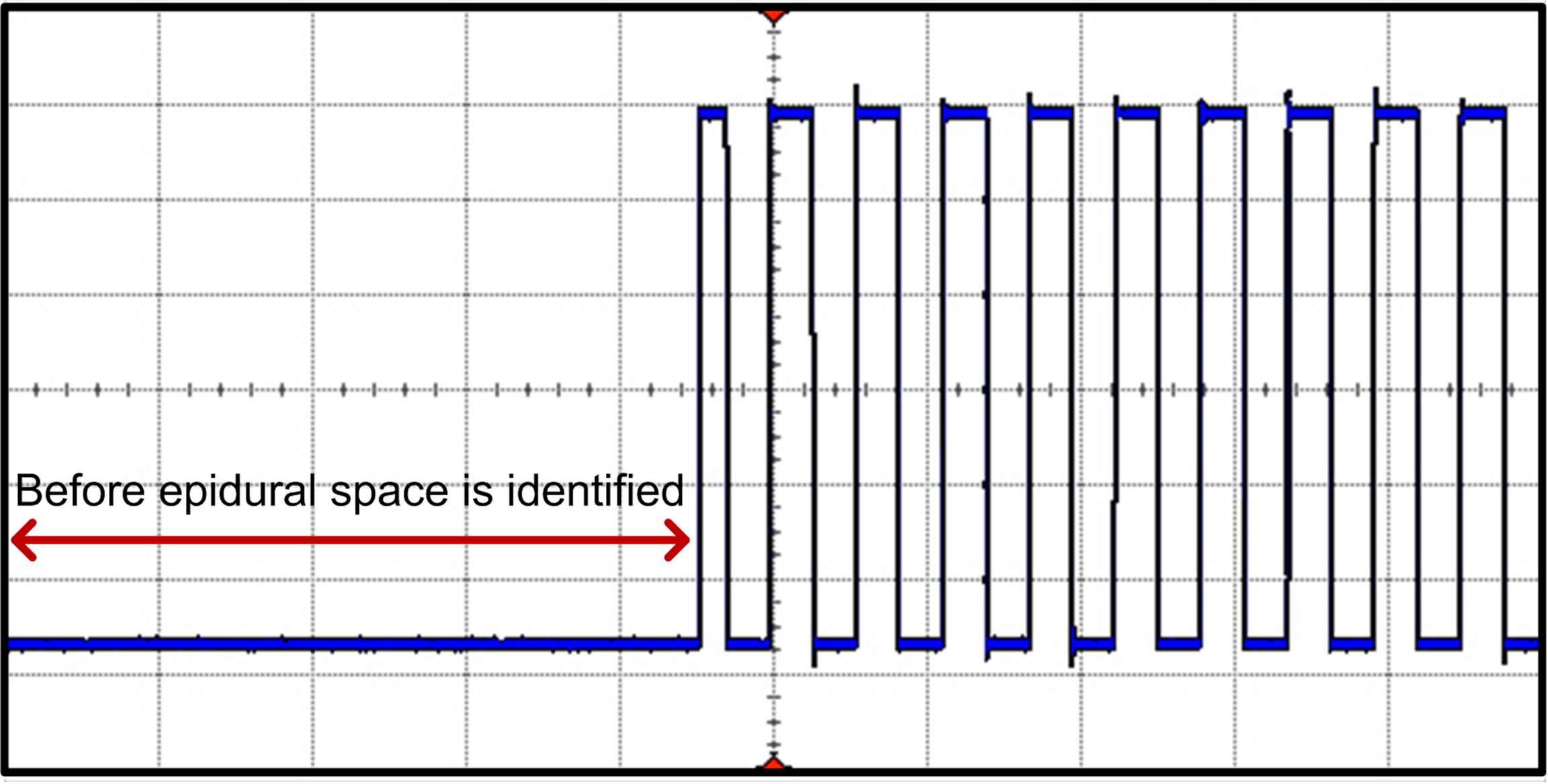

Supplement: Figure S8 — Measured buffered output of the Buzzer Driver input in an epidural space identification test. (TIF) [file pone.0106055.s008.tif]

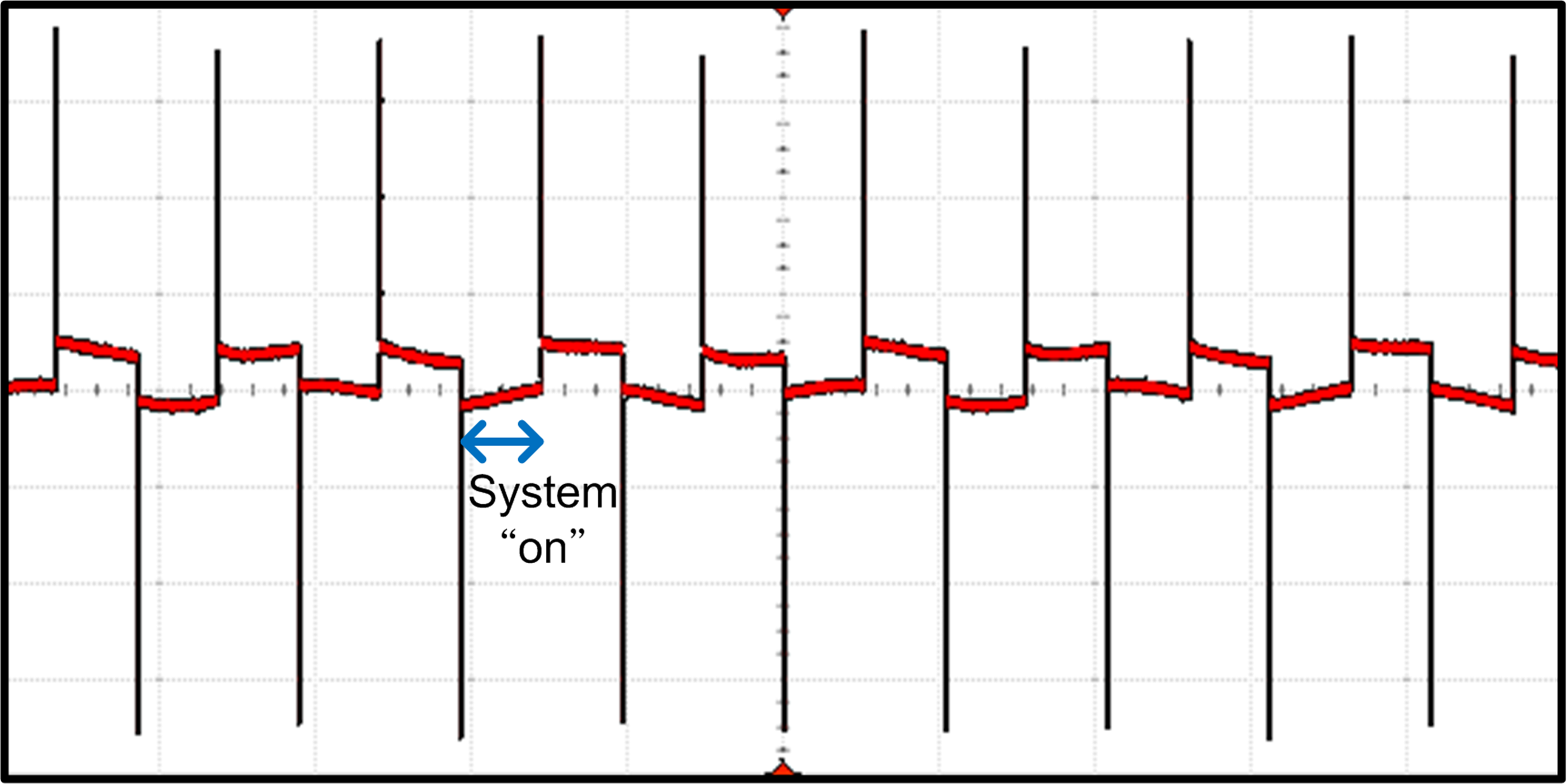

Supplement: Figure S9 — Measured regulator output performance of our ASD. (TIF) [file pone.0106055.s009.tif]
